# CoverageAnalyzer (CAn): A Tool for Inspection of Modification Signatures in RNA Sequencing Profiles

**DOI:** 10.3390/biom6040042

**Published:** 2016-11-04

**Authors:** Ralf Hauenschild, Stephan Werner, Lyudmil Tserovski, Andreas Hildebrandt, Yuri Motorin, Mark Helm

**Affiliations:** 1 Institute of Pharmacy and Biochemistry, Johannes Gutenberg University Mainz, Staudingerweg 5, 55128 Mainz, Germany; stwerner@uni-mainz.de (S.W.); ltserovs@uni-mainz.de (L.T.); 2 Institute for Computer Sciences, Johannes Gutenberg University Mainz, Staudingerweg 9, 55128 Mainz, Germany; Andreas.Hildebrandt@uni-mainz.de; 3 IMoPA UMR7365 CNRS-UL, BioPole de l’Université de Lorraine, 9 avenue de la Foret de Haye, 54505 Vandoeuvre-les-Nancy, France; motorine5@univ-lorraine.fr

**Keywords:** RNA modifications, reverse transcription, reverse transcription (RT) signature, RNA sequencing (RNA-Seq), Next-Generation Sequencing (NGS), candidate screening, alignment viewer

## Abstract

Combination of reverse transcription (RT) and deep sequencing has emerged as a powerful instrument for the detection of RNA modifications, a field that has seen a recent surge in activity because of its importance in gene regulation. Recent studies yielded high-resolution RT signatures of modified ribonucleotides relying on both sequence-dependent mismatch patterns and reverse transcription arrests. Common alignment viewers lack specialized functionality, such as filtering, tailored visualization, image export and differential analysis. Consequently, the community will profit from a platform seamlessly connecting detailed visual inspection of RT signatures and automated screening for modification candidates. CoverageAnalyzer (CAn) was developed in response to the demand for a powerful inspection tool. It is freely available for all three main operating systems. With SAM file format as standard input, CAn is an intuitive and user-friendly tool that is generally applicable to the large community of biomedical users, starting from simple visualization of RNA sequencing (RNA-Seq) data, up to sophisticated modification analysis with significance-based modification candidate calling.

## 1. Introduction

The detection of RNA modifications has recently re-emerged as a very timely topic of current research. Coupled to new detection methods came new insights into the function of RNA modifications in the regulation of RNA stability [1], regulation of gene expression [2,3,4,5], and immunity [6]. RNA modifications are structurally highly diverse, and among the approximately 150 chemically different structures in the Modomics database [7], all major classes of natural product compounds can be found [8,9]. Furthermore, there is evidence that the diversity may yet increase with the discovery of more modifications [10]. Despite this high diversity, some common denominators apply to both function and detection. Here, two important features for detection are reverse transcription (RT) arrest and misincorporation during complementary DNA (cDNA) synthesis. Before the advent of methods that are nowadays subsumed as deep sequencing, RT reverse transcription arrest was traditionally analyzed by gel or capillary electrophoresis [11]. A model modification for misincorporation, inosine, the product of an A-to-I deamination, is reliably reverse transcribed into a cytidine rather than a thymidine residue in the resulting cDNA. This misincorporation has led to the first transcriptome-wide mapping of an RNA modification [12]. The combined appearance of both RT arrest and misincorporation at modification sites was analyzed in early work [13,14]. Detailed analysis showed correlation between modification type and the relative composition of misincorporated nucleotides [15]. Also, chemical treatments that selectively alter the properties of a given modification [16,17] may therefore be exploited as an additional layer of information in single RNA species or in transcriptome-wide mapping [18,19,20]. Collection [7] and curation [21] of RNA sequences containing modifications underline a central problem in the field, arising from the vast number of candidate sites in large datasets. Because of these vast numbers, experimental verification of candidate sites by independent methods must typically be restricted to a small subset. Before engaging in such an endeavor, the experimentalist, and potential user of the software presented here, may want to assess the significance of an identification event, and visually inspect parameters at a given site. In principle, a variety of so-called alignment viewers like IGV, Tablet, Savant, UGENE and Persephone provide more or less detailed graphical representations of mapping results, typically resolving the base composition and orientation of reads covering a reference sequence. However, our recent application of machine learning approaches to the identification of modification sites has uncovered an unmet need for particular features in said tools. Specifically, the combination of mismatch patterns and a newly defined RT arrest rate has emerged as the central feature allowing efficient identification of 1-methyladenosine residues [22]. In response, CoverageAnalyzer (CAn) was specifically created for analysis of modification signatures in deep sequencing data. Distinct from variant caller and single nucleotide polymorphisms (SNP) identification tools, it allows the definition of a highly detailed query, based on combinations of arrest rates and mismatch composition, as well as a Context Sensitive Arrest rate (CSA). A differential visualization tool is particularly useful to compare signatures upon differential chemical treatment, or between wild-type and knockout mutants e.g., of a methyltransferase [22]. CAn combines a data processing pipeline with flexible controls for independent or differential visualization and automated screening for modification candidates based on complex RT signatures.

## 2. Results

**Figure 1:**
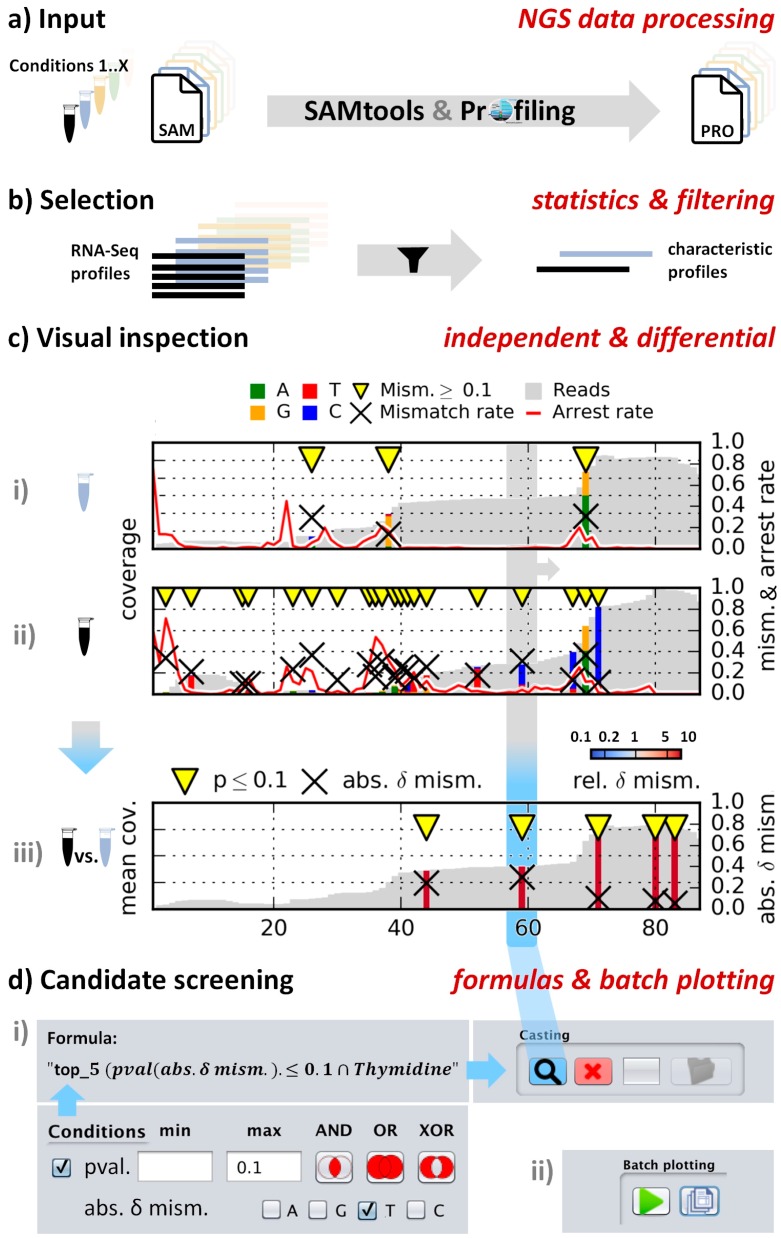
**Workflow for a typical CAn session** (**a**) Input SAM files are processed to a positional profile; (**b**) Sorting and filtering of data by various statistical criteria. From the depicted result table, users select sequences for visualization; (**c**) Visualization tab. Independent plots and differential comparison for mismatch and/or arrest parameters with marked above-threshold sites (yellow triangle). Display of base sequences is enabled automatically depending on the horizontal plot dimension; (**d**) Candidate casting tab; (**i**) Formula editor: Specification of screening thresholds. Conditions are combined with Boolean operators AND, OR and XOR and can be parenthesized; (**ii**) Control panel for serial plotting of a resulting candidate batch.

CAn was optimized to allow rapid pre-selection and convenient visualization of such sites in transcriptome data, which display conspicuous RT signatures and are therefore potential candidates for further scrutiny, e.g., by visual inspection. The RT signatures in question may comprise nucleotide misincorporation or transcription arrest, and frequently originate from nucleotide modification at the position of interest. Several library preparation protocols have been published that capture cDNA from abortive RT [16,20,22,23] and can therefore be fully exploited by CAn. However, even preparation methods that do not capture abortive cDNA may provide useful information by providing misincorporation signals that may be analyzed by CAn. It is hence recommended that the user familiarizes himself with details of the various preparations beforehand ([24]). A typical CAn-session, conceived to identify, highlight, and visually inspect modification candidates, is depicted in Figure 1. The user is required to input a dataset in SAM format, containing RNA sequencing (RNA Seq) reads mapped to a genome or transcriptome. This is converted to the internally used *Profile* format by an automated pipeline (Figure 1a). To optimally detect stalled RT events, a parameter called CSA was introduced, which queries a local background arrest rate near the inspection site and takes it into account. CSA was defined as the fold change of a site *i*’s arrest rate *A* [22] with respect to the median *A* of its sequence environment of *r* bases up- and *r* bases downstream (here *r* = 5):

(1) $$CSA^{r}\left(i\right)=\frac{A_{i}}{median\left(A_{i-5},A_{i-1},A_{i+1},\ldots ,A_{i+5}\right)}$$

The CSA feature, since it maps cDNA from abortive RT events, can only be meaningfully applied to data from library preparation protocols that specifically include such reads. Whether or not a protocol does so, typically hinges upon the incorporation step that introduces the second primer binding site. The installation package provides test datasets which were obtained by a library preparation protocol that captures abortive RT reads by ligation of a second adapter to the cDNA, as described in [22]. After selection of an RNA sequence of interest (Figure 1b), the software displays the sequence for visual inspection in a window (Figure 1c(i)). Events are labeled (yellow triangles), where values for misincorporation or peaks of CSA exceed adjustable thresholds. Additional profiles of the same sequence can be loaded and displayed in parallel plots (Figure 1c(ii)) for comparison of samples of variegated modification status, for example a wild-type RNA preparation *versus* one from a knockout organism lacking a certain modification activity [22]. Another application of interest is exemplified by the included test dataset, namely a chemical treatment suspected to alter the profile of certain modifications. With *test data 1* as the naive sample in window (i) and *test data 2* treated with an agent causing partial deamination of 5-methylcytidines in window (ii); a differential plot was generated in window (iii), where differences are displayed according to self-defined threshold criteria, and combinations thereof. The candidate casting tab (snippet shown in Figure 1d) offers a formula editor to generate filter rules of arbitrary complexity using thresholds combined by Boolean expressions and brackets. The resulting *candidates* files can be submitted to batch plotting for fast visual inspection of many candidate positions. With high flexibility in image dimensions, parameters, and legend details, these data can be exported as publication-ready images.

## 3. Discussion

CAn is a tool that allows the visualization and assisted inspection of deep sequencing data in the search for RNA modifications. Perusal of vast amounts of data is facilitated by a toolbox that allows to automatically highlight sites, where noticeably unusual combinations of RT arrest and misincorporation hint at the potential presence of modifications. Of note, there are no predefined thresholds that the program uses to flag unusual instances. Rather, it is up to the user to define threshold values for different parameters, and to combine them by Boolean operators. CAn is not meant to predict a modification event, or even to decipher the chemical structure of a potential modification. The program is rather designed to point attention to special candidate sites for its visual inspection. Inspection of large datasets automatically increases the statistical likelihood of the occurrence of conspicuous signals without a biochemical cause. Therefore, it is prudent to increase the stringency in such a case. While it is left to the user to decide how *p*-values are used to gauge the significance of findings, we recommend to use techniques like the Bonferroni correction [25] in order to account for the number of tested positions. In addition, the False Discovery Rate (FDR) can be controlled in the manner of Benjamini and Hochberg [26]. Outside these two approaches, which are rooted in statistics, a number of experimental approaches are open to the user to improve confidence by experimentally validating the candidate sites proposed by CAn. We urgently propose to call and treat these sites as “candidates” until validated by further experiments, e.g., by biochemical interrogation of a suggested site. In this context, we again emphasize the comparison feature, through which CAn specifically provides the possibility to inspect profiles before and after treatment with specific chemicals known to alter the RT-profile of a given modification. These may include, e.g., a Dimroth rearrangement of 1-methyladenosine (m^1^A) by alkaline treatment [27], acrylonitrile treatment for the detection of inosine [28], and others [16].

## 4. Materials and Methods

### 4.1. Implementation

The graphical user interface (GUI) and the core of CAn are written in Java. The Miniconda Python based plotting component uses Matplotlib [29], Numpy [30] and Scipy. The software is distributed as self-extracting archive (~100 MB) for Windows (64-bit) and as zip files installed via included script setup routines for Linux and Mac OS X. Dependencies are downloaded automatically. On Mac OS X, latest Homebrew is installed to setup SAMtools [31]. Java 1.7+ is expected to be installed by the user, whereas Linux version installs dependencies via *apt-get*. Test data, a getting-started screencast and a user manual are included.

### 4.2. Workflow

From unseen SAM input data files from *N* user-specified samples and the original FASTA mapping reference, CAn creates sorted and indexed Binary Alignment/Map (BAM) and finally the *Pileup* format. Users may replace the generated results on the hard drive with own files if they prefer different SAMtools parameters. In *Pileup*, periods and commas indicate matches, As, Gs, Ts and Cs mismatches and the arrest rate *A* of position *i* can be calculated as quotient of circumflexes at *i* + 1 and coverage at *i* + 1. Thus, a tabular *Profile* format is created, listing sequence positions line-wise with columns providing information on: position, reference base, coverage, mismatch rate *M*, number of (#) As, #Gs, #Ts, #Cs, and arrest rate *A*. *Profile* is divided into subfiles named by an x_y.txt tag, where *x* represents the reference number and *y* the y^th^ file of a 1 kb block of subsequence of reference *x*. For example, a file named 3_7.txt contains data for positions 6001–6430, if the third reference has 6430 nt. Hence, hashing allows fast access to a query region without reading or memorizing leading positions, when accessing ends of long reference sequences. Thus, although the scope is on short sequences of RNA, chromosomes can be handled, too. In parallel, statistics are gathered for reference sequences (Figure 1b): ID, file path, length, sequence (first 100 nucleotides (nt)), coverage peak, number of high-arrest sites (*S_A_*), high mismatch sites (*S_M_*), heterogeneous mismatch sites (*S_H_*) and mapped reads. This facilitates manual sorting and filtering by the user for visualization. Let *c* be the coverage at position *i* of reference *f* of length *n*. Let *R* be the reference base at *i*. Let $F_{b}\left(f,i\right)\frac{obs.\left(b,i\right)}{c\left(f_{i}\right)}:=$ where b ϵ{A,G,T,C} be the observed frequency of base type *b* covering *i* in *f*. Thus, $mF:=\left\{F_{b}\left(f,i\right),\mathrm{with}\;b\neq R\right\}$ is the set of mismatching $F_{b}\left(f,i\right)$. All *i* with c≥20 contribute to $S_{H_{f}}$, if two or more mismatch types exhibit a minimum mismatch rate of 0.1:

(2) $$S_{H_{f}}:=\overset{n}{\underset{i=1}{\sum }}x,\mathrm{where}\;x=1\;if\;c\left(f_{i}\right)\geq 20\;\mathrm{and}\;\underset{k}{\mathrm{median}}\;mF{\left(f,i\right)}_{k}\geq 0.1,\;0\;\mathrm{else}.$$

*S_A_* and *S_M_* are calculated similarly, for arrest or mismatch rates exceeding a threshold normalized with coverage *c*, such that low arrest rates are considered insignificant at low *c*, but captured if *c* is high.

## 5. Conclusions

CAn was developed as a cross-platform open-source software running on most current computers. It allows efficient inspection of RNA Seq profiles for RT signatures of modifications, such as m^1^A [22]. The user is provided with assistance to identify unusual patterns, to compare different datasets containing the same sequence, and to perform significance-based candidate calling. Important to the field is the implementation of both misincorporation patterns and RT arrest, including also the CSA format as defined during our recent extraction of RT signatures by machine learning [22]. CAn is highly conductive to the extraction of complete RT signatures, by providing full control of all thresholds for visualization, identification and discrimination to the user.

## Supplementary Materials

The following are available online at www.mdpi.com/2218-273X/6/4/42. CoverageAnalyzer setup files (incl. source code), instructions and documentation can be downloaded at: https://sourceforge.net/projects/coverageanalyzer/.
